# Supplementary material for: Complexome profiling of the Chlamydomonas psb28 mutant reveals TEF5 as an early PSII assembly factor
Source: Plant Cell. 2025 Mar 17;37(6):koaf055. doi: 10.1093/plcell/koaf055 (PMC12164590; doi:10.1093/plcell/koaf055)
Supplement: koaf055_Supplementary_Data [file koaf055_supplementary_data.zip › psb28_interactive.zip!/plots/intensity/qualityPlots/peptides/ETF2_Cre12.g554953.t1.1.html]

### Cre12.g554953.t1.1

Trivial name: ETF2  
SeqCoverage [median]: 6.90  
Mass: 28.69 kDa  
  
 

| Sequence | MissedCleavage | Mass [Da] | wtIntensity | muIntensity | Score |
| --- | --- | --- | --- | --- | --- |
| VGSVEELVRR | 1 | 1142.64 | 14618.03 | 0.00 | 44.20 |
| VIDYAVPVR | 0 | 1030.58 | 0.00 | 2175.89 | 66.68 |
|  |  |  | 14618.0 | 2175.9 |  |
